# Inhibition of Rho Kinase Regulates Specification of Early Differentiation Events in P19 Embryonal Carcinoma Stem Cells

**DOI:** 10.1371/journal.pone.0026484

**Published:** 2011-11-30

**Authors:** Roman J. Krawetz, Jaymi Taiani, Alexis Greene, Gregory M. Kelly, Derrick E. Rancourt

**Affiliations:** 1 Department of Surgery, University of Calgary, Calgary, Canada; 2 Department of Biochemistry and Molecular Biology, University of Calgary, Calgary, Canada; 3 Department of Medical Science, Faculty of Medicine, University of Calgary, Calgary, Canada; 4 Molecular Genetics Unit, Department of Biology, University of Western Ontario, London, Canada; Baylor College of Medicine, United States of America

## Abstract

**Background:**

The Rho kinase pathway plays a key role in many early cell/tissue determination events that take place in embryogenesis. Rho and its downstream effector Rho kinase (ROCK) play pivotal roles in cell migration, apoptosis (membrane blebbing), cell proliferation/cell cycle, cell-cell adhesion and gene regulation. We and others have previously demonstrated that inhibition of ROCK blocks endoderm differentiation in embryonal carcinoma stem cells, however, the effect of ROCK inhibition on mesoderm and ectoderm specification has not been fully examined. In this study, the role of ROCK within the specification and differentiation of all three germ layers was examined.

**Methodology/Principal Findings:**

P19 cells were treated with the specific ROCK inhibitor Y-27623, and increase in differentiation efficiency into neuro-ectodermal and mesodermal lineages was observed. However, as expected a dramatic decrease in early endodermal markers was observed when ROCK was inhibited. Interestingly, within these ROCK-inhibited RA treated cultures, increased levels of mesodermal or ectodermal markers were not observed, instead it was found that the pluripotent markers SSEA-1 and Oct-4 remained up-regulated similar to that seen in undifferentiated cultures. Using standard and widely accepted methods for reproducible P19 differentiation into all three germ layers, an enhancement of mesoderm and ectoderm differentiation with a concurrent loss of endoderm lineage specification was observed with Y-27632 treatment. Evidence would suggest that this effect is in part mediated through TGF-β and SMAD signaling as ROCK-inhibited cells displayed aberrant SMAD activation and did not return to a ‘ground’ state after the inhibition had been removed.

**Conclusions/Significance:**

Given this data and the fact that only a partial rescue of normal differentiation capacity occurred when ROCK inhibition was alleviated, the effect of ROCK inhibition on the differentiation capacity of pluripotent cell populations should be further examined to elucidate the role of the Rho-ROCK pathway in early cellular ‘fate’ decision making processes.

## Introduction

Rho proteins, which include Rho, Rac1, and Cdc42, control numerous cellular processes, such as cell adhesion, motility, proliferation, differentiation, and apoptosis [1]–[4]. One of the most well-defined effectors of Rho is the Rho-associated coiled-coil-containing protein kinase (ROCK). The ROCK inhibitor Y-27632 has recently attracted the attention of stem cell researchers as this molecule enhances the survival of human embryonic stem (hES) cells during dissociation [5]. Y-27632 inhibits ROCK by competing with its ATP binding site [6]. This in turn affects signal transduction in the Rho pathway, which perturbs downstream effects including the regulation of cytoskeletal integrity, cell adhesion and gene transcription [1]–[4], [7].

In the current study we used the P19 teratocarcinoma cell line as a model system to study the effects of ROCK inhibition on multi-lineage differentiation capability. P19 cells were derived from an embryonal carcinoma (EC) that was induced in a C3H/He strain mouse over 20 years ago [8]. These cells are pluripotent and thus possess the ability to differentiate into cells from all three germ layers [9]–[11]. Like other EC cells, P19 cells appear to differentiate using the same mechanisms as normal embryonic stem cells and will contribute to normal embryonic development when injected into mouse embryos [12]. Reproducible and validated differentiation protocols are established for P19 cells and these methods have had widespread usage with highly reproducible results [12]. For example, exposure to retinoic acid (RA) and leukemia inhibitory factor (LIF) induces P19 cell differentiation into neuronal and glial cells [13]. Also, endoderm derivatives are generated from P19 cells when they are treated with RA [12], whereas aggregates of P19 cells differentiate into cardiac and skeletal muscle in the presence of dimethyl sulfoxide (DMSO) [12]. This ability to develop into a disparate number of cell types therefore places the P19 cell line in a favourable position to study the role of RhoA in the differentiation process(es).

We and others have demonstrated that endoderm differentiation of mouse P19 and F9 EC cells requires the activation of Gα13 and Rho [14]–[18]. However, the role of ROCK within early cell fate decisions to mesoderm and endoderm has not been elucidated. To address this, the effects of blocking ROCK activity using the Y-27632 inhibitor were assessed on the ability of P19 cells to differentiate into specialized cell types from all three germ layers. Overall, it was observed that inhibition of ROCK activity by specific inhibitors altered the differentiation potential of pluripotent cells. In particular, this inhibition promoted P19 cell differentiation into mesodermal and ectodermal fates while reducing endodermal differentiation. Furthermore, Y-27632 treatment during directed differentiation into endodermal lineages resulted in an up-regulation of pluripotent markers with no indication of spontaneous differentiation into other lineages.

## Results

### Retinoic acid induced differentiation is inhibited by Y-27632

Endodermal differentiation using a static monolayer culture of P19 cells, and ectodermal and mesodermal differentiation with suspended aggregates of P19 cells was carried out as described previously [11]–[15]. To assess the effect of ROCK inhibition on differentiation, P19 cells were treated with the ROCK inhibitor Y-27632 and then induced to form endoderm or mesoderm and ectoderm.

**Figure 1:**
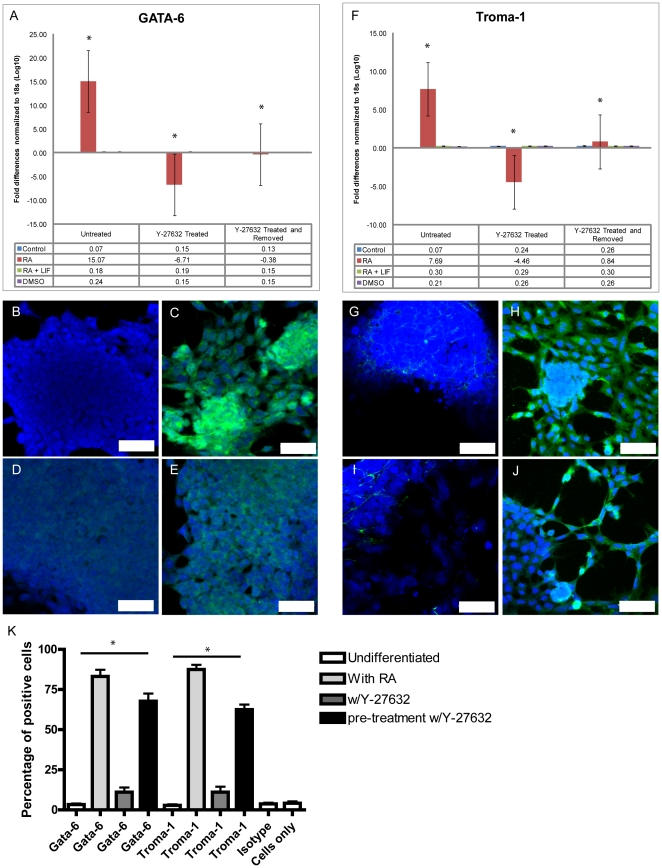
Y-27632 treatment of P19 cells inhibits the differentiation of primitive endoderm. Monolayer grown P19 cells induced to differentiate with RA were collected for mRNA quantification and immunofluorescence. Total mRNA from all treatment groups was probed with a GATA-6 primer/probe, which is a marker of primitive endoderm. The RA treatment group displayed an increase of GATA-6, but decreased when RA was supplemented with Y-27632, with moderate recovery when cells where induced to differentiate after Y-27632 pre-treatment and removal of the inhibitor (A). Immunofluorescence of GATA-6 in undifferentiated cells (B) and in RA treated (C), RA+Y-27632 treated (D) or RA+Y-27632 pre-treated cells. Total mRNA converted to cDNA and probed with Troma-1, showed a dramatic decrease in expression when Y-27632 was present either during or before differentiation (F). Immunofluorescence of Troma-1 in undifferentiated (B) RA treated (C), RA+Y-27632 treated (D) and RA+Y-27632 pre-treated cells. FACS analysis demonstrates a significant decrease in GATA-6 and Troma-1 positive cells with Y-27632 present (K). Scale bars equals 50 µm, green staining represents the primary antibody, and blue staining is the nuclear dye TOTO-3. * Significance accepted at p<0.05.

Extraembryonic primitive endoderm formation, induced in P19 cultures with the addition of RA, was accompanied by an increase in GATA-6 gene expression (Figure 1A). However, when Y-27632 was added to RA-treated cultures, GATA-6 expression was similar to that observed in the Y-27632-treated, undifferentiated cultures (Figure 1A). Interestingly, when P19 cells were pre-treated with Y-27632 for 4 days and then allowed to recover, GATA-6 expression increased, but did not recover to the same levels seen in untreated cells. Immunofluorescence analysis was also used to identify markers of differentiation in untreated and Y-27632-treated cells (Figure 1B–E). Undifferentiated P19 cells did not express GATA-6 (Figure 1B), whereas those exposed to RA showed GATA-6 staining in the nucleus (Figure 1C). Nuclear GATA-6 expression was not observed in RA and Y27632-treated cells (Figure 1D), but was present in RA-treated cells following the removal of the ROCK inhibitor (Figure 1E). The intensity of staining, however, was not to the extent as that observed in the controls. The expression of Troma-1, a marker of primitive endoderm, was also examined using qPCR (Figure 1F). Like GATA-6 gene expression, Troma-1 mRNA levels were significantly decreased in RA-induced endodermal cultures treated with Y-27632 relative to cells treated with RA alone. A partial rescue in Troma-1 expression also occurred when cells were allowed to recover from the inhibitor before being exposed to RA (Figure 1F). As expected, Troma-1 staining was weak in undifferentiated P19 aggregates (Figure 1G), whereas RA treatment induced prominent staining (Figure 1H). As with GATA-6, Troma-1 staining following Y-27632 treatment (Figure 1I) was similar to that in undifferentiated cells. Following the removal of the inhibitor, the reappearance of few Troma-1-positive filaments in RA-induced cells paralleled that seen for GATA-6 (Figure 1J). Finally, cells were dissociated and FACS analysis was used to identify the number of cells expressing either GATA-6 or Troma-1 (Figure 1K). RA treatment resulted in approximately 80% of the cells being positive for GATA-6 or Troma-1, whereas this number dropped to 12–15% when the ROCK inhibitor was present. Removing the inhibitor and allowing the cells to recover before endodermal induction resulted in approximately 65% of the cells being positive for GATA-6 and Troma-1 (Figure 1K). This data thereby corroborates the results seen from the two other independent analyses. That no significant difference in the mRNA level of GATA-6 or Troma-1 in cultures where ectodermal or mesodermal differentiation was induced by RA+LIF or DMSO, respectively, demonstrates the specificity of the differentiation protocol (Figure 1A,F). Together, these results would suggest that RhoA activity in P19 cells is required for endodermal differentiation.

### Differentiation of P19 into mesoderm or neural-ectoderm is enhanced by ROCK inhibition

**Figure 2:**
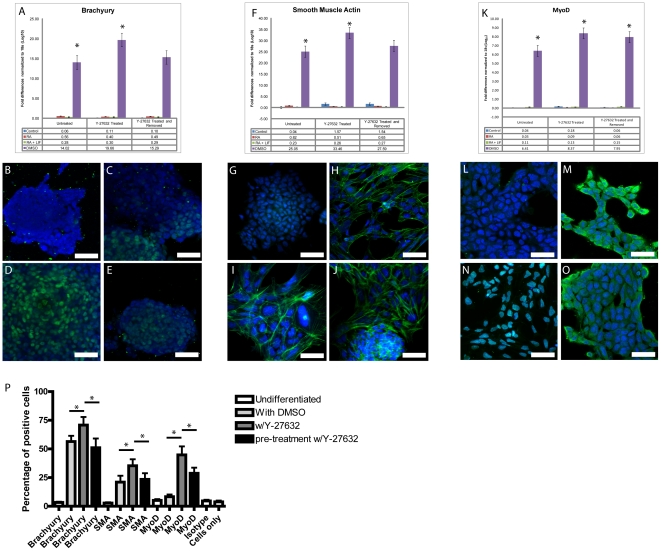
Differentiation of P19 cells into mesoderm is enhanced by Y-27632 treatment. P19 aggregates were treated with 1% DMSO to induce differentiation into mesoderm. Total mRNA was collected and probed with Brachyury, an early mesodermal marker. P19 cells treated with DMSO displayed an increase in Brachyury expression compared to cells treated with Y-27632 (A). Immunofluorescence analysis of cells either undifferentiated (B) or treated with DMSO treated (C) DMSO+Y-27632 treated (D) or DMSO treatment with Y-27632 pre-treatment (E) showing Brachyury localized to the nucleus. qPCR results for SMA show expression levels had increased following DMSO+Y-27632 treatment (F). Immunofluorescence of SMA in undifferentiated (G) DMSO treated (H) DMSO+Y-27632 treated (I) or DMSO treatment with Y-27632 pre-treatment (J). MyoD mRNA expression also increased with Y-27632 treatment in the presence of DMSO (K). Immunofluorescence of MyoD in undifferentiated cells (L) and in those treated with DMSO (M) DMSO+Y-27632 treated (N) or DMSO with Y-27632 pre-treatment (O). FACS analysis demonstrates a significant increase in Brachyury, SMA and MyoD positive cells with Y-27632 treatment (P). Scale bar equals 50 µm, green staining represents the primary antibody, and blue staining is the nuclear dye TOTO-3. * Significance accepted at p<0.05.

Mesodermal differentiation of P19 cells was induced using DMSO. When P19 cell aggregates were exposed to DMSO, mRNA levels of the mesoderm-specific transcription factor Brachyury increased dramatically as did smooth muscle Actin (SMA) and MyoD (Figure 2A). The relative levels of Brachyury, after pre-treatment with Y-27632 and removal of the inhibitor, were not significantly different from control DMSO-treated cells (Figure 2A), and no obvious Brachyury staining was found in undifferentiated P19 cells (Figure 2B). The induction of mesoderm was accompanied by the appearance of Brachyury in the nuclei of a sub-population of cells (Figure 2C), whereas when cells were treated with DMSO in the presence of Y-27632, almost every cell exhibited nuclear Brachyury staining (Figure 2D). Brachyury-positive cells were also observed in those pre-treated with Y-27632 and then with DMSO (Figure 2E). This staining pattern was similar to that in the DMSO-treated cells.

The muscle markers SMA and MyoD were also examined and their patterns of expression compared to that for Brachyury. SMA actin mRNA levels were significantly up-regulated following Y-2632 treatment (Figure 2F). No actin fibres were observed in undifferentiated cells (Figure 2G), however, multiple actin fibres were seen following DMSO induction (Figure 2H). When Y-27632 and DMSO were added together, SMA fibres formed and the nuclei appeared larger (Figure 2I). Pre-treatment with Y-27632 followed by DMSO produced similar results (Figure 2J). Quantitative PCR analysis revealed that a significant up-regulation in MyoD mRNA levels was observed following DMSO treatment (Figure 2K). At the protein level, MyoD was not detected in undifferentiated P19 cells (Figure 2L), but was seen in the cytoplasm and nucleus of cells induced with DMSO (Figure 2M). It is interesting to note that MyoD was observed exclusively in the nuclei of cells that were exposed to Y-27632 while in the induction media (Figure 2N). The pattern of MyoD staining in pre-treated cells (Figure 2O) was similar to that in cells treated with DMSO alone. Finally, FACS analysis of cells stained for Brachyury, SMA or MyoD revealed an interesting trend. Results showed that for each of the markers, those cells exposed to DMSO and Y-27632 demonstrated a higher percentage of positive staining compared to DMSO alone or Y-27632-pre-treated cells (Figure 2P).

**Figure 3:**
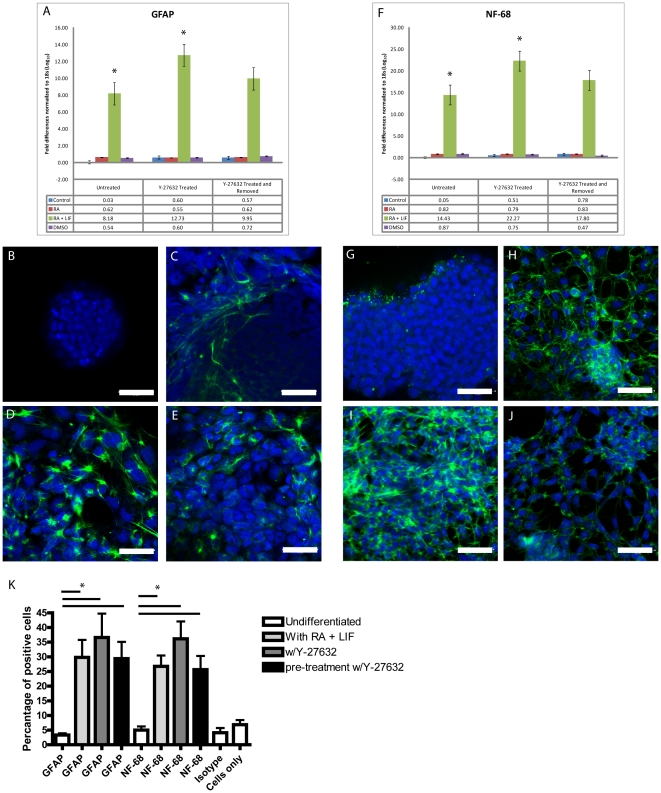
Y-27632 enhances the differentiation of P19 cells into ectoderm. P19 cell aggregates were treated with RA+LIF to induced neural-ectoderm differentiation. Total mRNA was collected, converted to cDNA and used in a PCR to examine GFAP expression, a marker of ectodermal derived neural cell types (A). GFAP levels increased with RA+LIF treatment in the presence or absence of Y-27632. Increased GFAP expression was also detected in the DMSO treated groups supplemented with Y-27632. Immunofluorescent detection of GFAP in undifferentiated (B) RA+LIF (C) RA+LIF and Y-27632 (D) or RA+LIF with Y-27632 pre-treated cells (E). qPCR with NF-68 primers showed an increase in expression in cells exposed to Y-27632 during differentiation (F). Immunofluorescent detection of NF-68 in undifferentiated cells or those exposed to the following: (G) RA+LIF (H) RA+LIF and Y-27632 (I) or RA+LIF with Y-27632 pre-treatment (J). FACS analysis demonstrates increased numbers of GFAP- (trend) and NF-68-positive cells with Y-27632 treatment during RA+LIF induced differentiation (K). Scale bar equals 50 µm, green staining represents the primary antibody, and blue staining is the nuclear dye TOTO-3. * Significance accepted at p<0.05.

To better understand if ROCK has a role in early ectodermal differentiation, P19 cell aggregates were exposed to RA & LIF to block endodermal and mesodermal differentiation [15]. Glial fibrillary acidic protein (GFAP) and NF-68 were used as markers of neuro-ectodermal differentiation. Quantitative PCR results showed that GFAP mRNA expression, relative to the undifferentiated cultures, increased in cells following exposure to RA+LIF (Figure 3A). Moreover, these levels increased when the RA+LIF treatment was combined with Y-27632 (Figure 3A). Immunofluorescence microscopy was also used to identify changes in protein expression. Results did not show any obvious GFAP staining in undifferentiated P19 aggregates (Figure 3B), whereas GFAP-positive cells were present at the periphery of the aggregates when cells were exposed to RA+LIF (Figure 3C). Dramatic morphological changes to these ectodermal cells occurred when they were treated with Y-27632 (Figure 3D). The most obvious changes included the appearance of more GFAP-positive cells, the absence of cell aggregates and the change in individual cell morphology. The fact that these changes were maintained when the inhibitor was removed (Figure 3E), suggested that attenuating Rho activity augmented the signaling required by cells to adopt an ectodermal fate.

Immunofluorescence using an antibody against NF-68, which recognizes neuro-filaments, was used to further test this hypothesis that blocking Rho activity influenced the decision of cells to form neuro-ectoderm. Results show that NF-68 mRNA levels increased in cells treated with RA+LIF and these levels were significantly higher when cultures were exposed to Y-27632 (Figure 3F). Immunofluorescence data showing extensive NF-68 staining in RA+LIF-treated P19 cells (Figure 3H), but not in undifferentiated cells (Figure 3G) corroborates the qPCR analysis. Likewise, NF-68 staining was even more prominent in ectodermal cells exposed to the ROCK inhibitor (Figure 3I), and this trend in staining seen using the other markers was once again maintained when the inhibitor was removed (Figure 3J). Finally, data from FACS analysis of GFAP and NF-68 positive cells were consistent with the mRNA and immunofluorescence data (Figure 3K). The number of GFAP (although not significantly) and NF-68 positive cells increased when differentiated cells were exposed to Y-27632, however, the relative percent of each declined if the inhibitor was removed and the cells had time to recover before the induction of differentiation (Figure 3K).

### Signal transduction and pluripotency is regulated by ROCK

**Figure 4:**
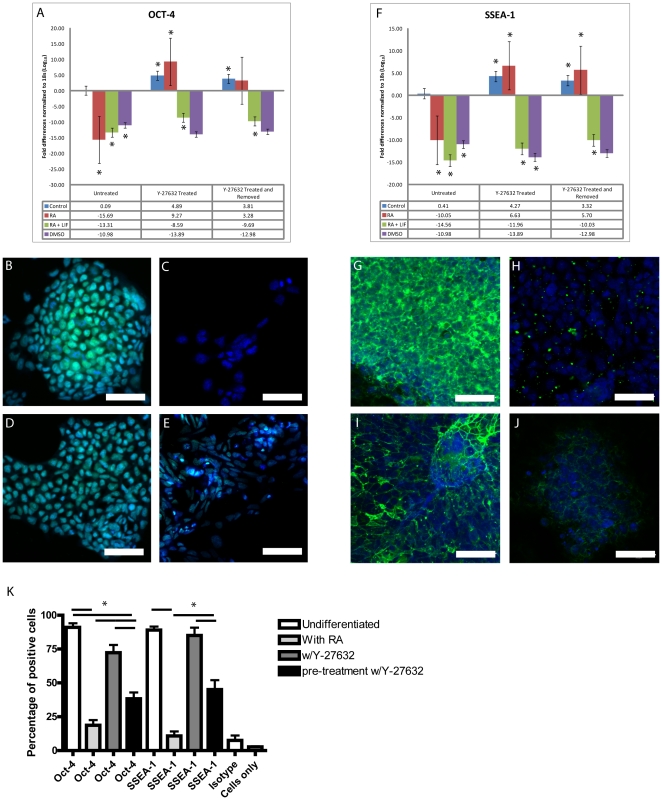
The decrease in endoderm differentiation observed in RA+Y-27632 treatment corresponds to maintenance of pluripotency. Total mRNA collected from samples was used in a qPCR with primers against the pluripotency marker Oct-4 (A). As expected, during directed differentiation into all three germ layers, the expression of Oct-4 is decreased. However, under RA+Y-27632 treatment (A) Oct-4 levels are more similar to that in undifferentiated cells. Immunofluorescent detection of Oct-4 in undifferentiated cells (B) and in cells treated with RA (C) RA plus Y-27632 (D) or RA with Y-27632 pre-treatment (E). qPCR with SSEA-1 probes showed similar levels and trends to that of Oct-4, with significant decreases observed during differentiation into any lineage (F). Furthermore, as with Oct-4 SSEA-1 mRNA expression remained at control levels during RA with Y-27632 treatment (F). Immunofluorescent detection of SSEA-1 in undifferentiated cells (G) and in those treated with RA (H) RA with Y-27632 (I) or RA with Y-27632 pre-treatment (J). FACS analysis demonstrates increased numbers of Oct-4 and SSEA-1 positive cells during RA induced differentiation and Y-27632 treatment (K). Scale bar equals 50 µm, green staining represents the primary antibody, and blue staining is the nuclear dye TOTO-3. * Significance accepted at p<0.05.

Data would indicate that blocking ROCK activity negatively affects P19 cells from differentiating into endoderm, positively affects the differentiation towards mesodermal and ectodermal lineages, or a combination of both. The next series of experiments were designed to investigate whether or not blocking ROCK activity affects pluripotency of these EC cells. Oct-4 and SSEA-1 are markers of pluripotency and their expression was examined in undifferentiated and differentiated P19 cultures. Quantitative PCR analysis confirmed that differentiation into endoderm, ectoderm or mesoderm triggered a decrease in Oct-4 and SSEA-1 expression compared to that in undifferentiated cultures (Figure 4A,F). Furthermore, the addition or pre-treatment with Y-27632 had no affect on the declining Oct-4 and SSEA-1 levels seen in cells induced to form ectoderm (RA+LIF) or mesoderm (DMSO) (Figure 4A,F). Interestingly, Oct-4 and SSEA-1 levels remained elevated when cells were treated with RA in the presence of Y-27632 (Figure 4A,F). In contrast, however, cells pre-treated with the inhibitor and allowed to recover did not show this trend and Oct-4 and SSEA-1 levels remained low. When the localization of Oct-4 was examined by immunofluorescence microscopy, staining was seen in the nuclei of untreated cells (Figure 4B), but absent when the cells differentiated into endoderm (Figure 4C). RA induced cultures treated with Y-27632 showed obvious Oct-4 staining throughout the cell and this was accompanied by dramatic changes in cellular morphology (Figure 4D). Y-27632 pre-treated cells allowed to recover, before being induced with RA, exhibited similar changes in cell morphology, but Oct-4 staining was weak (Figure 4E).

**Figure 5:**
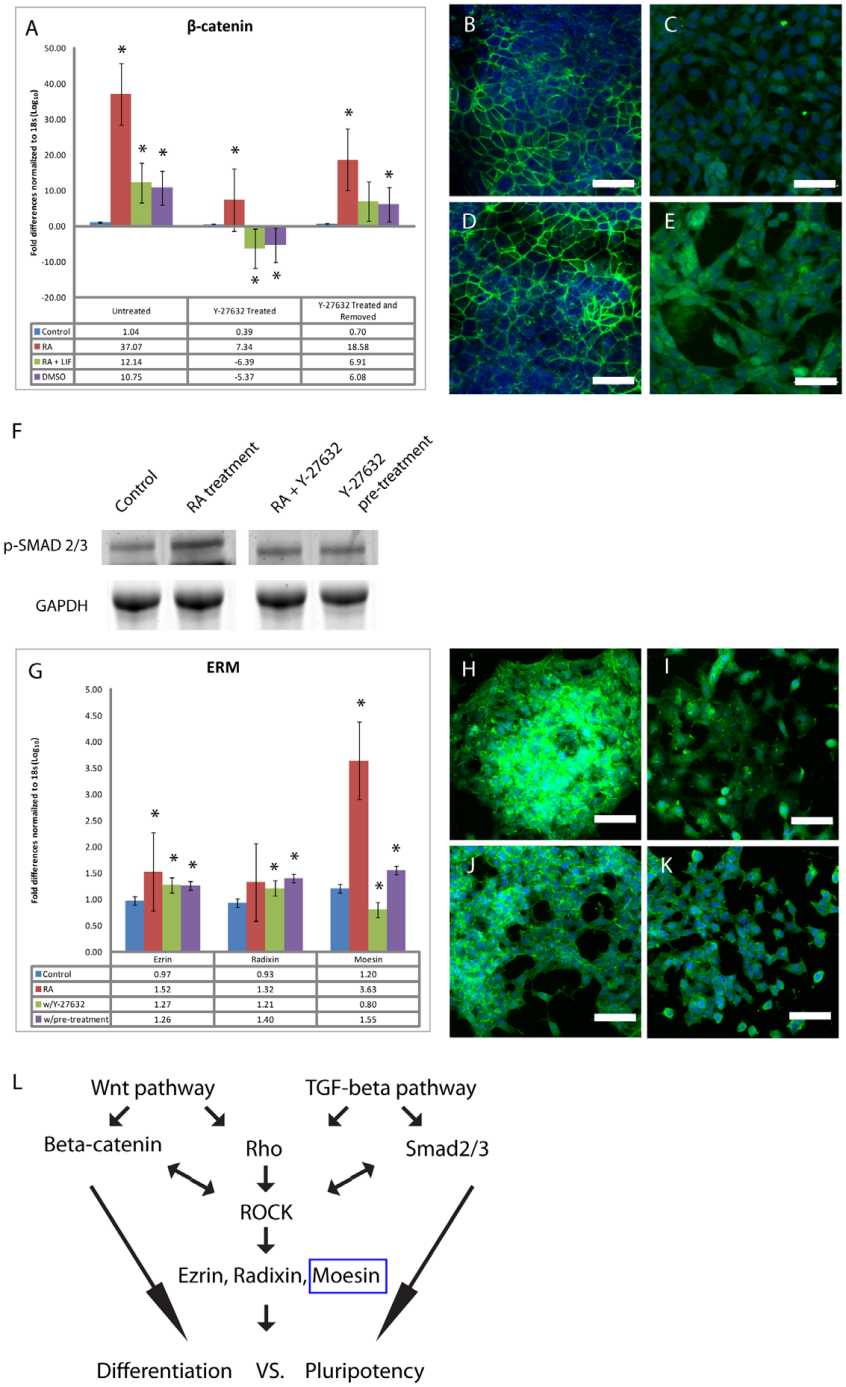
Y-27632 mediated signal transduction during differentiation. Total mRNA collected from all groups was converted to cDNA and probed to assay levels of β-catenin (A). β-catenin levels increased under all differentiation induction conditions, however, levels decreased significantly when Y-27632 was added during mesodermal and ectodermal differentiation. Immunofluorescent detection of β-catenin in undifferentiated cells (B) and cells treated with RA (C) RA plus Y-27632 (D) or RA with Y-27632 pre-treatment (E). To assay the activity of the TGF-β pathway, cell lysates were probed with a phospo-SMAD2/3 antibody (F). Results demonstrate that a significant decrease in SMAD activation occurred when Y-27632 was present or after pre-treatment and removal of the inhibitor. cDNA from RA induced cells was assayed for Ezrin, Radixin and Moesin expression levels (G). A pan-ERM antibody was used to stain undifferentiated cells (H) and those exposed to RA (I) RA plus Y-27632 (J) or RA with Y-27632 pre-treatment (K). Model representing ROCK interaction with multiple pathways influencing the ‘fate’ of unspecified stem cells (L). Scale bar equals 50 µm, green staining represents the primary antibody, and blue staining is the nuclear dye TOTO-3. * Significance accepted at p<0.05.

SSEA-1 expression showed a similar trend as observed for Oct-4. Positive staining was seen on differentiated P19 cells (Figure 4G), but absent on those induced with RA (Figure 4H). SSEA-1 was also expressed on RA-induced cells treated with Y-27632 (Figure 5I), and like Oct-4, staining was still visible, albeit weak, on cells pre-treated with the inhibitor and then induced with RA (Figure 4J). FACS analysis corroborated the immunofluorescence data suggesting that pluripotency was enhanced when Rho signaling was attenuated (Figure 4K). Results showed few Oct-4 and SSEA-1-positive cells when endoderm was induced, whereas the majority of cells were positive for both markers when the ROCK inhibitor was present, or after it was removed (Figure 4K). Together, these results would support the notion that Rho activity is required for cells to adopt an endodermal lineage because it lifts the restrictions required to keep cells in a pluripotent state.

To help elucidate the mechanism(s) behind these observations, attention turned to signal transduction pathways involved in regulation of both pluripotency and differentiation. β-catenin is involved in the patterning of all three germ layers during early mouse development and it participates in numerous pathways required for the regulation of differentiation. Recent studies in human embryonic stem cells have demonstrated that ROCK inhibition blocks cadherin proteins from undergoing ‘membrane flipping’ [19]. This flipping normally releases β-catenin from the plasma membrane and allows it to translocate into the nucleus. Furthermore, we have also shown that nuclear translocation of β-catenin accompanies F9 endodermal differentiation [14]. To extend these studies, the levels of β-catenin mRNA expression were examined in differentiating P19 cells in the presence or absence of Y-27632. Results show that levels were comparable between undifferentiated P19 cultures and those treated with DMSO or RA+LIF (Figure 5A). There was, however, a statistically significant increase in β-catenin levels during RA-induced differentiation (Figure 5A), which was expected given our previous report [14]. Although β-catenin levels in RA-induced cells were negatively affected by Y-27632 treatment (Figure 5A), they were still higher than those in untreated, undifferentiated cells. The opposite, however, was seen in cells induced with DMSO or RA+LIF in the presence of Y-27632, where the levels were significantly lower than those in the controls. It is noteworthy that this decrease was not as dramatic in cells pre-treated with the inhibitor and then induced towards a specific lineage. Having determined that β-catenin levels were influenced either directly or indirectly by ROCK activity, the focus turned to the sub-cellular distribution of β-catenin since the targets of β-catenin signaling are not only influenced by the level of β-catenin expression, but also by the localization of this protein [14], [16]–[18]. We and others have demonstrated that β-catenin translocation to the nucleus accompanies the differentiation of F9 cells into primitive endoderm under RA treatment and although β-catenin is expressed in undifferentiated F9 cells, it is found primarily at the plasma membrane [14], [16]–[18]. Immunofluorescence microscopy revealed that β-catenin was also expressed at the plasma membrane of undifferentiated P19 cells (Figure 5B). Similarly, β-catenin translocated into the nucleus when P19 cells were induced to form endoderm, (Figure 5C). In RA treated P19 cells Y-27632 blocked the nuclear translocation of β-catenin, and staining was localized to the plasma membrane (Figure 5D). If, however, cells were allowed to recover from ROCK inhibition, then treated with RA, β-catenin staining was seen in the nucleus (Figure 5E). Together, the results would suggest that blocking Rho activity promoted pluripotency, but alleviating this block thereby facilitating the translocation of β-catenin to the nucleus, promotes differentiation towards the endodermal lineage.

Having identified a requirement for Rho activity and canonical β-catenin signaling during differentiation of P19 cells towards specific lineages, the last pathway we considered was that involving TGF-β. The TGF-β pathway is involved in cellular differentiation and in the maintenance of stem cell pluripotency through the regulation of Nanog and Oct-4 expression [20], [21]. In some cases TGF-β signals directly through Rho and ROCK, and inhibition of ROCK blocks the effects observed when TGF-β is added to the system [22], [23]. Involvement of the TGF-β pathway was explored by probing cell lysates for phosphorylated SMAD2/3, whose presence would be indicative of active TGF-β signaling (Figure 5F). Immunoblot analysis showed that the levels of active p-SMAD 2/3 increased with RA treatment, but surprisingly, the levels in cells treated with RA and Y-27632 were similar to those in the controls (Figure 5F). Furthermore, when cells were first treated with the inhibitor and then given time to recover, p-SMAD 2/3 levels did not increase, but instead they remained comparable to the controls (Figure 5F). This data would indicate that ROCK may play a role in the activation of SMAD 2/3 independent of TGF-β.

Having shown that Rho signaling acting through ROCK influences two signaling pathways involved in P19 differentiation, the next logical question was to investigate the effectors downstream of this ROCK activity. ROCK is known to act on numerous substrates including the members of the ERM (Ezrin, Radixin and Moesin) protein family [24]. Our previous studies with F9 cells have shown that Moesin is essential for both extraembryonic endodermal differentiation and survival [14], which leads us to propose that a similar requirement may exist for P19 cells. To address this, the expression profile of Moesin mRNA as well as the sub-cellular localization of the protein was examined in P19 cells treated with RA in the presence or absence of Y-27632. Results show that Moesin mRNA levels increased significantly during RA induction (Figure 5G), but surprisingly this expression was inhibited in cells exposed to Y-27632. In untreated cells immunofluorescent analysis with a pan-ERM antibody revealed a relatively homogenous cytoplasmic staining pattern with some foci present within the colonies (Figure 5H). Although cells treated with RA still showed these positive foci as well as staining at the cell membrane, staining could also be seen within nuclei, which we reported previously for F9 cells [20] (Figure 5I). Significantly, the addition of Y-27632 blocked this nuclear staining even though positive signals were still seen in the cytoplasm (Figure 5J). Pre-treating cells with Y-27632 before RA induction resulted in cells having both staining patterns (Figure 5K). Together, these results would suggest that the Moesin redistribution to the nucleus in RA-treated P19 cells was not unlike that seen in F9 cells, and that this re-localization is dependent on ROCK activity. The necessity of Moesin for extraembryonic endoderm differentiation remains to be determined, but it is easy to envisage a model whereby ROCK activity not only facilitates the phosphorylation and subsequent translocation of Moesin to the nucleus, but in parallel this activity is required to negatively regulate the TGF-β signaling required to keep ES cells pluripotent (Figure 5L).

## Discussion

Induced differentiation of P19 cells has been characterized extensively and it is well known that in the absence of chemical inducers, these cells rarely differentiate spontaneously [12]. In contrast, exposure of P19 cells to RA leads to formation of endodermal cells [9]–[11]. Likewise, neuro-ectodermal lineages, including neurons and astroglial cells, are generated when P19 cells are aggregated and then exposed to RA and LIF [9]–[12]. Finally, mesodermal lineages, including cardiac and skeletal muscle, are formed when P19 aggregates are treated with DMSO. Interestingly, however, is that aggregation of P19 cells in the absence of chemical inducers results in the differentiation of the cells into predominantly endodermal lineages, signifying that cell-matrix interactions maintains the P19 cells in an undifferentiated state (in the absence of inducers), whereas cell-cell contact without cell-matrix interactions can in part substitute for RA [9]. Under all of these conditions, LIF inhibits differentiation of P19 cells towards endodermal or mesodermal lineages, but does not inhibit differentiation of neuro-ectodermal lineages. Thus, treatment with this cytokine contributes to a higher efficiency of ectodermal differentiation when P19 cells are concurrently treated with high doses of RA [13]. Cell aggregation is essential for P19 cell neural differentiation, while over-expression of RA-induced genes triggers P19 cell neural differentiation from within these aggregates [25]–[27].

We and others have demonstrated the necessity of cell-cell/cell-matrix adhesion for the proper maintenance and differentiation of stem cells [28], [29]. Furthermore, the extensive intracellular remodelling that takes place during differentiation is accompanied by extensive changes in gene expression. Rho-associated protein kinases (ROCKs) are downstream effectors of Rho GTPase, which function in numerous physiological processes including the reorganization of cytoskeletal elements [30]–[32]. In addition to this role, our results would indicate that ROCK inhibition blocks the differentiation of endoderm, whereas it also attenuates the induction of P19 cells into mesoderm or ectoderm, but only in the presence of DMSO or RA and LIF, respectively. If the ROCK inhibitor is directly affecting endoderm differentiation by interfering with RA-induced gene regulation, we would expect both endoderm and ectoderm differentiation to be affected by Y-27632 since they both rely on being triggered by RA. However, this is not the case, as RA and LIF in conjunction with ROCK inhibition leads to an increase in GFAP and NF-68 expression, implying that ROCK itself must be instrumental only in endoderm differentiation of P19 EC cells.

Restriction of endodermal differentiation through ROCK inhibition may result from altered signaling through the ERM proteins, which are regulated by ROCK. Normally, under Rho activation, ROCK phosphorylates the ERM proteins and this leads to remodelling of the actin cytoskeleton [33]. We have previously demonstrated that the over-expression of Moesin influences EC cells to differentiate into primitive endoderm in the absence of chemical inducers [34]. In the present study, we have demonstrated that Moesin mRNA levels are up-regulated during RA induced differentiation, and Y-27632 treatment represses this effect. Therefore, we believe that ectodermal and specifically neuro-ectoderm differentiation through RA treatment may activate signaling pathways that diverge somewhere before the level of Rho and ROCK. As such, the capacity of P19 cells to differentiate into ectodermal lineages would not be affected by ROCK inhibition, supporting our observations in the current study. Alternatively, ROCK may be functioning within a model by which multiple signal transduction pathways are not only responsible for the maintenance of pluripotency, but are also essential for differentiation into specific cell types of all three germ layers. In this case, where pluripotency vs. differentiation is controlled by the same or similar factors, one would expect that a strict balance must be maintained between numerous signals. As an example, the literature shows that Rho and ROCK participate with the β-catenin and TGF-β signaling pathways, even though the TGF-β or Wnt proteins are absent [35]–[36]. In other cases ROCK acts downstream when Wnt and TGF-β are present [37]. Therefore, we suggest that ROCK has the ability to serve as an intermediate between these pathways, possibly controlling this balance between active/inactive downstream states (Figure 5L). This may either be direct, as ROCK inhibition can lead to the loss of SMAD phosphorylation [22], or indirect, in the case of ROCK stabilizing β-catenin within the cadherin complex [19].

It is also important to recognize that the Rho-ROCK pathway is essential for maintenance and re-organization of actin stress fibers and focal contacts, which can result in changes in cell motility and morphology [31]. These changes are of importance as cell-cell and cell-ECM interactions are fundamental processes involved in cell migration, differentiation and tissue remodelling. Not surprisingly, the inhibition of Rho or ROCK leads to a decrease in cell-cell interactions and increased migration [31]. In relation to this study, one would expect that decreased cell-cell interactions during aggregate formation would impede ectoderm differentiation [38], however, the opposite effect was observed. Aggregation of P19 cells in the presence of Y-27632 did not completely abolish cell adhesion, but the aggregates did not appear as ‘tightly’ packed as in controls (data not shown). While previous studies have demonstrated that cell-cell interactions are essential for differentiation into ectoderm [38], we observed increased expression of ectodermal and mesodermal markers when ROCK is inhibited. One explanation may be that cells exhibiting a decreased level of cell adhesion, but remaining in a three dimensional structure may be the optimal condition for P19 ectodermal or mesodermal differentiation, possibly through something as simple as increased diffusion of inducing factors. Interestingly, a similar effect was not seen for endodermal differentiation. The differentiation of primitive endoderm, described as the earliest epithelial-to-mesenchymal transition in the developing mouse embryo, requires an increase in migration and decrease in cell-cell adhesion to occur under normal conditions [39]. In some cases increased migration and decreased adhesion have been directly observed under Y-27632 treatment [40], however, differentiation of endoderm is not solely dependent on these changes. In fact, Y-27632 treatment of P19 cells completely abolished the endoderm inducing effect of RA. As we and other groups have described, the differentiation of EC into endoderm in a multi-factorial process that not only involves crosstalk between signal transduction pathways, but also the modification of the cytoskeleton [14]–[18], [34]. We therefore suggest that ROCK is playing many roles within the cell and that by inhibiting ROCK, certain elements of differentiation are enhanced. At the same time other aspects of differentiation may have a high requirement for activation of the Rho-ROCK signaling axis, which of course would have negative consequences when ROCK is inhibited.

Overall, we have demonstrated that ROCK is playing a role in early fate determination of stem cells. Furthermore, it appears that ROCK may be acting through SMAD proteins to affect changes in gene expression in conjunction with other signal transduction pathways (canonical Wnt signaling) to determine which fate (ecto/endo/mesoderm) an unspecified cell will adopt. ROCK interacting with SMADs and other pathways was reported previously [22]. Given the recent reports to indicate that ROCK inhibition has significant effects on the differentiation of human embryonic stem cells [41], [42], our data would suggest that ROCK itself may act as a lynch-pin in early fate determination in pluripotent stem cells.

## Materials and Methods

### Cell Culture

Mouse P19 cells (ATCC) were plated on 0.1% gelatin coated 35 mm dishes for protein isolation or coverslips for immunofluorescence, and grown at 37°C and 5% CO_2_ in Dulbecco's Modified Eagle's Medium (DMEM/F-12; Invitrogen) supplemented with 10% fetal bovine serum (FBS; Invitrogen), 1% non-essential amino acids (Invitrogen), β-mercaptoethanol (Invitrogen) and 1% penicillin-streptomycin (Invitrogen). To induce differentiation, aggregates containing approx. 1000 cells were formed using the hanging drop method. Aggregates were harvested after 24 hours and replated in medium supplemented with either 10^−7^ M retinoic acid (RA, all-trans; Sigma) plus 1000units/ml of LIF (Calbiochem) for ectoderm differentiation or 1% DMSO (Sigma) for mesoderm differentiation. For endoderm differentiation, cells in monolayer were treated with 10^−7^ M RA. Cells were cultured in differentiation medium for 4 days before being harvested for analysis. All treatments were carried out in the presence or absence of 10 µM ROCK inhibitor Y-27632 (Sigma). For Y-27632 experiments cells were cultured in the presence of 10 µM of the inhibitor for 4 days and then the inhibitor was replaced with fresh media for an additional 4 days before differentiation was induced. All experiments were carried out at least 3 times using similar passage P19 cells (passages 6–10).

### Protein Analysis

At the end of each experiment cells were lysed in sodium dodecyl sulfate loading buffer containing phenylmethylsulfonyl fluoride (PMSF; Sigma). Extracts were collected and centrifuged at 15,000× g for 15 min at room temperature and supernatants stored at −20°C. Protein concentration was quantified using the Bradford method (Bio-Rad), and then separated on a 10% polyacrylamide gel. The gel was then transferred to nitro-cellulose membrane. The membrane was blocked with 3% skim milk powder in Tris-buffered saline (TBS) for 1 hr at room temperature. After blocking, the membrane was probed for 2 hr at room temperature with the following antibodies: GAPDH (Santa Cruz), p-SMAD 2/3 (Santa Cruz), all using a 1∶2000 dilution in Tris buffer saline containing Tween 20 (0.5%) (TBST) and supplemented with skim milk powder (3%). The membrane was then washed three times in TBST and blocked for 1 hr in 3% skim milk powder in TBST. The corresponding secondary antibody conjugated to an infra-red emitter (LI-COR) was added to fresh blocking solution and incubated with the membrane for 2 hr at room temperature. The membrane was washed three times in TBST and scanned with the Odyssey infra-red system (LI-COR).

### Statistical analysis

Statistical analysis was performed using GraphPad Prisim (v. 4) software utilizing a 1-way ANOVA and a Bonferroni post test.

### Immunofluorescence

Cells grown on coverslips were fixed in phosphate buffered saline (PBS) containing 4% paraformaldehyde, then washed in PBS and permeabilized with 0.1% Triton X-100 for 10 min. After washing in PBS, cells were incubated for 20 min in 3% bovine serum albumin (BSA) to reduce non-specific protein-protein interactions. Cells were incubated at 37°C for 1 hr in the appropriate primary antibody at dilutions of 1∶50 in 3% BSA [GFAP (NEB), β-catenin (DSHB), GATA-6 (Santa Cruz), SSEA-1 (DSHB), Brachyury (Santa Cruz), NF-68 (Santa Cruz), p-SMAD 2/3 (Santa Cruz) and Oct-4 (Santa Cruz)]. Following incubation, cells were washed in PBS and then treated with 3% BSA for 20 min before being incubated for 1 hr at 37°C with the appropriate AlexaFlor488-conjugated secondary antibody (Molecular Probes; 1∶100) and TOTO-3 (Invitrogen) to stain the nucleus. Cells were given a final wash in PBS, then mounted on slides using 9∶1 PBS: glycerol and sealed with nail polish. Slides were analyzed using a Zeiss 510 confocal microscope with a 488 nm filter, and images prepared with Zeiss LSM image browsing software.

### Flow Cytometry

Cells obtained from at least 3 independent experiments were stained using the primary antibodies described above. Primary antibodies were directly conjugated to Alexa Fluor 488 using Zenon conjugation kits (Invitrogen) following manufacturer's instructions. The single cell suspension was subjected to fluorescence-activated cell counting using a Calibur instrument and the CellQuest software from Becton Dickinson. Anti-mouse, goat or rabbit secondary antibodies were used as isotype controls.

### Quantitative Polymerase Chain Reaction (qPCR)

RNA was collected using Trizol (Invitrogen) and converted to cDNA using a capacity kit (Applied Biosystems (ABI), Carlsbad, CA). The cDNA was probed using validated Taqman primer/probe-sets for murine GFAP, β-catenin, GATA-6, SSEA-1, Brachyury, SMA, MyoD, NF-68, Ezrin, Radixin, Moesin and Oct-4 on an ABI 7900HT using 18S as the internal control and the ddCT method included within the ABI software to analyze results.
